# Desidustat for the Treatment of Anaemia in Adult End-Stage Kidney Disease Patients on Maintenance Haemodialysis: A Prospective Observational Study

**DOI:** 10.1155/ijne/5375585

**Published:** 2025-10-16

**Authors:** Ankit Tiwari, Niranjan M., Karthik Kalidindi, Siddharth Herur, Anshul Dudani, Vijay Chander Bukka, Gangadhar Taduri, Swarnalatha Guditi

**Affiliations:** Department of Nephrology, Nizam's Institute of Medical Sciences, Hyderabad, India

**Keywords:** anaemia, chronic kidney disease, desidustat, end-stage kidney disease, ESA, haemoglobin

## Abstract

**Aim:**

Anaemia is a prevalent complication in patients with end-stage kidney disease (ESKD), significantly impacting morbidity and mortality. Desidustat, a hypoxia-inducible factor prolyl hydroxylase inhibitor, offers an alternative to erythropoiesis-stimulating agents for managing anaemia. This study aims to assess the response of haemoglobin and safety and efficacy of desidustat in ESKD patients.

**Methods:**

This prospective observational study evaluated the efficacy and safety of desidustat in 50 ESKD patients on maintenance haemodialysis. Patients received 100 mg oral desidustat thrice weekly for 6 months, with regular monitoring of haemoglobin, iron profiles, lipid profiles, and adverse effects.

**Results:**

This study demonstrated a 76% response rate to desidustat, with a mean haemoglobin rise of 0.82 g/dL (9.56% increase) over 6 months. Notably, higher baseline serum iron and ferritin levels predicted greater responses. Additionally, desidustat reduced serum ferritin, TSAT, LDL, and total cholesterol levels without impacting blood pressure.

**Conclusion:**

Desidustat demonstrated efficacy and safety in ESKD patients on haemodialysis, offering effective haemoglobin management and a favourable safety profile. These findings support desidustat as a viable treatment option for patients with anaemia, providing a valuable alternative to existing therapies.

**Summary**

• This prospective study evaluated oral desidustat in 50 ESKD patients on haemodialysis.
• Desidustat showed a 76% response rate with significant haemoglobin rise, improved iron indices, and reduced cholesterol levels without affecting blood pressure.
• It demonstrated efficacy and safety and offers a promising alternative for anaemia management in dialysis-dependent CKD patients.

## 1. Introduction

Anaemia is a hallmark of advanced kidney disease, characterized by decreased haemoglobin, haematocrit, and erythrocyte count, leading to significant morbidity, mortality, and disease progression. In chronic kidney disease (CKD), anaemia results from factors like shortened red cell lifespan, functional iron deficiency, diminished erythropoietin (EPO) production, and EPO resistance [1]. Erythropoiesis-stimulating agents (ESAs) are commonly used to control anaemia, but hypoxia-inducible factor (HIF) activation offers an alternative approach. HIF-PH inhibitors, such as desidustat, stimulate erythropoiesis, improve iron metabolism, and reduce hepcidin synthesis [2]. Recently approved in India for CKD-related anaemia, desidustat has shown efficacy and safety comparable to epoetin alfa, with no increased risks [3]. This study was conducted to evaluate the efficacy and safety of desidustat in the native population, providing a valuable treatment option for CKD patients on dialysis with anaemia.

## 2. Methods

It was a prospective observational study conducted on ESKD patients on MHD at Nizam's Institute of Medical Sciences (NIMS), Hyderabad, from January 2023 to January 2024. All adult patients aged 18–60 years with haemoglobin between 7 and 10 g/dL were included. Patients having iron, folate, and/or B12 deficiency, past history of EPO intake (less than 12 weeks), history of blood transfusion less than 8 weeks, history of kidney transplant, haemoglobin < 7 g/dL, patients who do not wish to continue in the study and special groups like pregnant woman, lactating women, children < 18 years, elderly > 60 years, differently abled (mental/physical), terminally ill (stigmatized or rare diseases), refugee and migrants were excluded.

### 2.1. Study Procedures

After getting due consent from Institutional Ethics Committee, baseline demographic, clinical characteristics, and laboratory parameters like anaemia profile (B12, serum folate, serum iron, TSAT, % saturation and serum ferritin) and lipid profile were collected from eligible patients. Iron deficiency was excluded based on TSAT and serum ferritin levels. After ruling out nutritional cause of anaemia, with TSAT > 20% and serum ferritin > 200 mg/dL, patients were enrolled in the study. All the enrolled patients received 100 mg oral desidustat given thrice weekly off haemodialysis days for convenience. Monthly systolic and diastolic BP was measured before HD on nonfistula arm via oscillatory BP machine. Monthly change in haemoglobin, and iron profile (serum ferritin and % saturation), and quarterly change in lipid profile were assessed for 6 months. Side effects, if any, were monitored on every visit. Any patient with serious adverse events was stopped from continuing the drug but was followed for 6 months.

### 2.2. Statistical Analysis

The % change in haemoglobin level and mean haemoglobin change from the baseline were assessed. Response was assessed as haemoglobin rise of > 0.5 g/dL during 6 months. It was further graded as mild response if haemoglobin rise was between 0.5 g/dL to 1 gm//dL and moderate response if haemoglobin rise was more than 1 gm/dL. At any time, if target Hb of more than 11 g/dL was attained, drug was stopped. After the end of the study, i.e., 6 months, all the data were analysed in SPSS software.

Frequency and percentage analysis were used for categorical variables and mean ± SD for continuous variables. To find significant differences between paired groups, the paired sample *t*-test was used. For multivariate analysis of repeated measures, repeated measures ANOVA was performed with Bonferroni correction to control type I error on multiple comparisons. The assumption of sphericity was checked using Mauchly's test; where sphericity was violated, Greenhouse–Geisser correction was applied. A *p*-value < 0.05 was considered statistically significant.

### 2.3. Results and Observations

Total number of patients studied was 50. Mean age was 43 ± 11.5 years with male predominance.

Male:Female ratio = 1.8:1. Most patients (84%) were hypertensive, and 24% patients were diabetic. Other morbidity includes coronary artery disease and hypothyroidism. There were diverse causes of kidney disease, most common being diabetic kidney disease (24%), chronic glomerulonephritis (22%), and chronic interstitial nephritis (18%), followed by other autoimmune and hypertensive conditions, and least was reflux nephropathy (2%) (Table 1). 58% patients had past history of taking ESA therapy; however, only those were included, who stopped ESA > 3 months prior to recruitment (Table 1).

A total of 38 (76%) out of 50 patients demonstrated improved haemoglobin levels, with 10 patients (26.3%) achieving a substantial increase of over 1 g/dL and 28 patients (73.7%) experiencing a mild rise of 0.5–1 g/dL. Importantly, these responders demonstrated sustained and statistically significant monthly Hb increase throughout the study duration of 6 months. When comparing responder's vs nonresponders, higher baseline serum iron and ferritin levels were associated with a rise in haemoglobin. Other parameters like TSAT, ongoing iron therapy, serum B12 level, serum folate levels, baseline CRP levels, gender, and native kidney disease had no influence. A comparison within responder (*n* = 38) revealed that individuals with a haemoglobin increase of > 1 g/dL had higher baseline serum iron and total iron binding capacity (TIBC) levels compared to those with mild responders (0.5–1 g/dL). This suggests that higher baseline serum iron and TIBC levels are associated with a greater rise in Hb (Table 2). A significant decline in serum ferritin and TSAT was observed over 6 months. Additionally, LDL and total cholesterol (TC) levels demonstrated significant reductions at 3 and 6 months. In contrast, no significant changes were observed in systolic and diastolic blood pressure, VLDL, HDL, and triglyceride levels over the six-month period (Table 3). Regarding adverse effects, 26% patient did not have any adverse effects. Among remaining 74%, gastrointestinal symptoms were most common side effect namely nausea/vomiting (16%). Acute decompensated heart failure (ADHF), lower limb cellulitis, and CRBSI of tunnelled catheters were critical side effects found in 2% of patients each, requiring hospitalization, but were managed conservatively.

## 3. Discussion

### 3.1. Effect on Haemoglobin

In our study, out of 50, 38 patients (76%) showed response, and there was increase in mean haemoglobin of 0.82 g/dL. None of patient achieved target haemoglobin level (11 g/dL), but there was 9.56% increase in the haemoglobin levels over 6 months. In most of the other studies involving patients on dialysis (Table 4), the mean change in haemoglobin was less than 1 g/dL, similar to the findings in our study, however one study [4] showed haemoglobin rise of 2.57 ± 0.4 g/dL while, in other studies [7, 8], the haemoglobin change was negative. Indian studies [9, 10] on CKD predialysis and dialysis patients showed significant improvement in Hb level postbaseline of more than 1 gm/dL. In a meta-analysis by Chen et al. [11], there were no differences with desidustat group in the mean change of the haemoglobin level from baseline, but recent meta-analysis [12] showed that desidustat was more frequently associated with target haemoglobin achievement as compared to other agents. These findings suggest that while desidustat may not significantly impact mean haemoglobin changes, it might help patients in maintaining target haemoglobin levels.

### 3.2. Effect on Iron Profile

Our study revealed decrease in ferritin levels and TSAT as seen in other studies [3, 13–17]. Our study revealed a significant correlation between baseline iron indices and haemoglobin response. Responders had higher baseline serum iron and ferritin levels compared to nonresponders and within the responder subgroup, and those achieving > 1 g/dL rise in haemoglobin had higher baseline serum iron and TIBC. Interestingly, baseline TSAT was not significantly different between responders and nonresponders, and TSAT levels decreased progressively over the 6-month period across all groups. This pattern suggests that absolute iron stores (ferritin) and circulating iron availability (serum iron), rather than TSAT, may be more predictive of responsiveness to desidustat therapy in dialysis patients.

A possible explanation for these findings is that desidustat enhances erythropoiesis by mobilizing stored iron and improving iron utilization efficiency. Patients with higher baseline serum iron and ferritin may therefore have greater substrate availability to support red cell production, leading to a more robust haemoglobin response. The observed decline in TSAT over time could reflect increased iron consumption by active erythropoiesis, reinforcing the importance of adequate baseline iron stores when initiating therapy. These results highlight the role of baseline iron status in predicting response and suggest that optimizing iron stores before desidustat initiation may improve treatment outcomes.

The mechanism in iron metabolism can be explained in terms of increase in iron in circulation, increase in iron absorption and efficient iron utilization, and promoting the utilization of stored iron. This does not mean that it will aggravate iron deficiency, but efficient erythropoiesis and hence rise in haemoglobin [15]. Thus, in addition to increasing endogenous EPO production, it reduces the need for intravenous (IV) iron supplementation [17].

### 3.3. Effect on Lipid Profile

In our study, there was a significant decrease in LDL and TC levels but no changes in HDL or VLDL levels similar to our other studies [3, 13, 14, 18]. The LDL cholesterol lowering effect may be mediated by HIF-dependent effects on acetyl coenzyme A that are required for the first step of cholesterol synthesis, and on the degradation of 3-hydroxy-3-methylglutaryl coenzyme-A reductase, the rate-limiting enzyme in cholesterol synthesis. This reduction in TC and LDL cholesterol may be a benefit, because dyslipidemia and hypertension are risk factors for cardiovascular disease in patients with CKD [19]. Meta-analysis [12, 18] revealed a statistically significant decline in LDL levels; however, this reduction had limited practical implications for reducing cardiovascular-related illness and death. Notably, HDL levels remained stable in our study. Previous research indicates that HIF-PHIs may decrease HDL cholesterol levels, potentially mitigating the cardioprotective benefits [12]. Hence, more research is needed on the role of HIF-PHIs in regulating lipid metabolism and its benefits in dialysis patients.

### 3.4. Effect on BP

There was no difference in either systolic or diastolic blood pressure during the 24 weeks course. Various other studies including meta-analysis [3, 12–14, 19, 20] showed similar results.

### 3.5. Adverse Effects

In our study, 13 (26%) patients reported no adverse effects. The remaining 37 (74%) experienced mild to moderate and nonlife-threatening complaints. Notably, three patients required hospitalization for unrelated complications, catheter-related bloodstream infection (CRBSI) managed with antibiotics, accelerated hypertension with acute decompensated pulmonary oedema, resolved with medical treatment and lower limb cellulitis treated with oral antibiotics. Importantly, these complications were deemed unrelated to desidustat. Furthermore, no patients withdrew from the study or discontinued medication due to adverse effects. In other studies, including meta-analysis [3, 5, 6, 9–11, 13, 14, 17, 20, 21], there was no major adverse effect or life-threatening effect. Only mild-to-moderate adverse effects were reported like our study which was managed conservatively. In a meta-analysis [22], desidustat, when compared to ESA, had no difference in the risk of major adverse cardiovascular event (MACE), all-cause death and cardiovascular death. In our study, no death was reported.

A key strength of our study is its focus on the native South Indian population, offering valuable insights into the efficacy of desidustat within this specific demographic. The well-defined study population, consisting of ESKD patients on MHD, ensures clarity and specificity. Additionally, the comparative analysis with existing research enhances the study's relevance and generalizability. The limitations of our study were its small sample size and observational design.

## 4. Conclusion

This study demonstrated the efficacy and safety of desidustat in 50 CKD patients on haemodialysis, with 76% response rate and mean haemoglobin rise of 0.82 g/dL (9.56% rise) over six months. Notably, none of the patients achieved the target haemoglobin of 11 g/dL even after 6 months of therapy, suggesting that ESAs may still be required to achieve target Hb levels. However, desidustat may allow for easier long-term haemoglobin maintenance, potentially reducing ESA burden. These findings, together with its favourable safety profile, support desidustat as a valuable therapeutic option for anaemia management in dialysis patients.

## Figures and Tables

**Table 1:** Baseline characteristics.

Parameter	Values
Age (years), mean ± SD	43.0 ± 11.5
Gender M:F	1.8:1
BMI (kg/m^2^), mean ± SD	26.7 ± 4.0
HD vintage (months), mean ± SD	20.1 ± 9.1
Comorbidities hypertension, *n* (%)	42 (84)
DM, *n* (%)	12 (24)
CAD, *n* (%)	8 (16)
Hypothyroidism, *n* (%)	7 (14)
Others, *n* (%)	14 (28)
Access:Permanent (AVF), *n* (%)	45 (90)
Temporary (RIJVTC), *n* (%)	5 (10)
NKD CGN presumed, *n* (%)	11 (22)
CIN presumed, *n* (%)	9 (18)
DKD, *n* (%)	12 (24)
IgA, *n* (%)	5 (10)
LN, *n* (%)	4 (8)
Malignant hypertension, *n* (%)	5 (10)
TLC (/mm^3^), mean ± SD	7712.0 ± 1402.7
PLT (× 10^3^), mean ± SD	201.3 ± 32.3
Creatinine (mg/dL), mean ± SD	6.0 ± 1.4
UREA (mg/dL), mean ± SD	67.2 ± 16.6
SGOT (U/L), mean ± SD	18.7 ± 5.5
SGPT (U/L), mean ± SD	14.5 ± 4.1
Total bilirubin (mg/dL), mean ± SD	0.7 ± 0.2
TotalSerumProtein (g/dL), mean ± SD	6.9 ± 0.3
Albumin (g/dL), mean ± SD	3.7 ± 0.3
hsCRP (mg/L), mean ± SD	6.8 ± 3.9
B12 (pg/mL), mean ± SD	1167.2 ± 576.8
FOLATE (ng/mL), mean ± SD	12.2 ± 7.5

**Table 2:** Difference between responders vs nonresponders.

	Nonresponders	Responders	*p* value	Subgroup analysis within responders		*p* value
	Hb < 0.5 g/dL	Hb > 0.5 g/dL		> 1 g/dL	0.5–< 1 g/dL	
Number	12	38		10	28	
Mean iron (ug/dL)	50.9	64.315	0.014^∗^	76.2	50.7	0.012^∗^
Mean ferritin (ng/mL)	707.16	845.78	0.003^∗^	850.7	844.75	0.879
TSAT (%)	24.66	27.08	0.311	31.5	25.44	0.134
TIBC (ug/dL)	234.08	238.39	0.818	258.9	231.4	0.018^∗^
B12 (pg/mL)	889.1	978	0.998	871.5	862.7	0.385
FOLATE (ng/mL)	7.8	9.67	0.389	7.78	10.48	0.669
Male	10	21	0.100	5	16	0.152
Female	2	17		5	12	
DM %	6 (50%)	6 (15.8%)	0.16	3 (30%)	3 (10.7%)	2.061
HTN %	10 (83%)	3 (84.2%)	1.0	9 (90%)	23 (82.14%)	0.342
CAD %	3 (25%)	5 (13.15%)	0.379	2 (20%)	3 (10.7%)	0.556
NKD-CGN %	1 (8.33%)	19 (50%)	0.14	4 (40%)	15 (53.58%)	7.213
DKD %	6 (50%)	6 (15.8%)		3 (30%)	3 (10.7%)	
LN %	0	3 (7.9%)		1 (10%)	2 (7.14%)	
IgA %	1 (8.33%)	3 (7.9%)		1 (10%)	2 (7.14%)	
CIN %	1 (8.33%)	2 (5.26%)		1 (10%)	3 (10.7%)	
HTN %	3 (25%)	3 (7.9%)		0	3 (10.7%)	
Baseline CRP (mg/L)	7.47	6.8	0.889	5.27	6.5	1.21

^∗^statistically significant.

**Table 3:** Trends in haemoglobin levels, iron stores, blood pressure, and lipid profiles.

Variable	Baseline	1st month	2nd month	3rd month	4th month	5th month	6th month	*p* value
Haemoglobin (g/dL), mean ± SD	8.56 ± 0.64	8.70 ± 0.67	8.84 ± 0.60	9.0 ± 0.67	9.09 ± 0.70	9.23 ± 0.70	9.38 ± 0.73	0.0001^∗^
Ferritin (ng/mL), mean ± SD	812.5 ± 482.6	790.5 ± 486.7	765.9 ± 439.9	746.9 ± 415.2	728.2 ± 374.5	695.7 ± 373.0	674.8 ± 366.1	0.0005^∗^
TSAT (%), mean ± SD	27.1 ± 8.9	30.5 ± 7.1	29.4 ± 6.5	28.8 ± 6.3	28.8 ± 6.6	27.4 ± 7.0	26.1 ± 7.7	0.0005^∗^
Systolic blood pressure (mm hg), mean ± SD	139.3 ± 11.3	136.0 ± 11.7	137.0 ± 10.6	137.1 ± 12.6	137.1 ± 11.4	136.8 ± 10.3	136.6 ± 13.6	0.114
Diastolic blood pressure (mm hg), mean ± SD	88.4 ± 9.6	88.3 ± 8.1	88.0 ± 7.2	89.0 ± 7.4	88.7 ± 6.1	87.5 ± 5.6	86.5 ± 5.3	0.210
LDL (mg/dL), mean ± SD	79.7 ± 33.7	—	—	74.4 ± 30.1	—	—	69.9 ± 26.3	0.0005^∗^
VLDL (mg/dL), mean ± SD	29.0 ± 12.1	—	—	29.5 ± 12.6	—	—	28.3 ± 9.9	0.663
HDL (mg/dL), mean ± SD	38.4 ± 12	—	—	39.3 ± 11.1	—	—	40.5 ± 11.6	0.211
TGL (mg/dL), mean ± SD	125.0 ± 57.8	—	—	119.6 ± 66.2	—	—	113.5 ± 54.6	0.064
TC (mg/dL), mean ± SD	142.7 ± 44.6	—	—	133.2 ± 43.0	—	—	120.1 ± 33.8	0.0005^∗^

^∗^statistically significant.

**Table 4:** Comparison with previous studies.

S. no	Study	Type	*N*	Duration (weeks)	Dose	Drug	Control	Mean age (years)	Male/female (%)	Mean Hb (g/dL)	Difference in HB (g/dL)	% change
1.	HIMALAYAS 2021 [4]	RCT	522	52	70 mg < 70 kg 100 mg > 70 kg	Roxadustat	Epoetin	54.1	49/41	8.4	2.57	30% increase
2.	DIALOGUE 5, 2019 [5]	RCT	87	144	25–150 mg	Molidustat	Epoetin alfa/beta	60	69/31	10.4	0.5	4.8% increase
3.	INNO2VATE 2021 [6]	RCT	3554	116	300–600 mg	Vadadustat	Darbepoetin	58	47/43	10.7	0.23	2.2% increase
4.	DREAM-D 2022 [3]	RCT	196	24	100 mg	Desidustat	Epoetin	51.4	69/31	9.61	0.95	9.9% increase
5.	Our study	Cohort	50	24	100 mg	Desidustat	—	42.5	62/38	8.5	0.82	9.56% Increase

## Data Availability

The datasets generated and/or analysed during the current study are available from the corresponding author on reasonable request.

## Nomenclature

BMI: Body mass index
HD: Haemodialysis
DKD: Diabetic kidney disease
CGN: Chronic glomerulonephritis
CIN: Chronic interstitial nephritis
CAD: Coronary artery disease
HTN: Hypertension
DM: Diabetic mellitus
LN: Lupus nephritis
AVF: Arteriovenous fistula
NKD: Native kidney disease
HB: Haemoglobin
TSAT: Transferrin saturation
TIBC: Total iron-binding capacity
HDL: High-density lipoprotein
LDL: Low-density lipoprotein
VLDL: Very low-density lipoprotein
TGL: Triglycerides
TC: Total cholesterol
